# Mercury in Marine and Oceanic Waters—a Review

**DOI:** 10.1007/s11270-016-3060-3

**Published:** 2016-09-07

**Authors:** Barbara Gworek, Olga Bemowska-Kałabun, Marta Kijeńska, Justyna Wrzosek-Jakubowska

**Affiliations:** Institute of Environmental Protection–National Research Institute, Warsaw, Poland

**Keywords:** Mercury, Marine waters, Benthic sediments, Pollution, Environment

## Abstract

Mercury contamination in water has been an issue to the environment and human health. In this article, mercury in marine and oceanic waters has been reviewed. In the aquatic environment, mercury occurs in many forms, which depend on the oxidation-reduction conditions. These forms have been briefly described in this article. Mercury concentrations in marine waters in the different parts of the world have been presented. In the relevant literature, two models describing the fate and behavior of mercury in saltwater reservoirs have been presented, a conceptual model which treats all the oceans as one ocean and the “ocean margin” model, providing that the ocean margins manifested themselves as the convergence of continents and oceans, covering such geological features, such as estuaries, inland seas, and the continental shelf. These two conceptual models have been summarized in the text. The mercury content in benthic sediments usually reflects is level in the water reservoir, particularly in reservoirs situated in contaminated areas (mines, metallurgical plants, chemically protected crops). The concentrations of mercury and its compounds determined in the sediments in surface waters in the different parts of the world have been presented. Due to the fact that the pollution caused by mercury is a serious threat for the marine environment, the short paragraph about mercury bioaccumulation in aquatic organisms has been included. The cited data demonstrated a large scatter of mercury contents both between the fish species and the water areas. Mathematical models, valuable tools which provide information about the possible responses of ecosystems, developed to simulate mercury emissions, both at a small scale, for local water reservoirs, and at a global scale, as well as to model mercury bioaccumulation in the chain web of aquatic systems have been described.

## Introduction

Mercury is an element the emissions of which from its natural sources exceed its anthropogenic emissions. Therefore, it is difficult to estimate mercury emission levels from its natural sources. This is related, e.g., to the absence of sufficient data on this issue and the complexity of processes affecting natural mercury emissions (to mention only geological processes which are characterized by enormous spatial and temporal variabilities). In general, natural mercury emissions into the environment can be divided into original emissions, e.g., volcanic emissions, and secondary reemissions of natural origin. Natural mercury emissions can also be divided into emissions from oceans which are estimated at 2680 Mg/year and those of terrestrial origin, estimated at 1850 Mg/year [Mason 2009; Pirrone et al. 2010; Pirrone and Cinnirella 2012) with volcanic emissions to the atmosphere estimated as 94–112 Mg/year (Nriagu and Becker 2003; Nriagu and Becker 2004). Natural mercury emission processes mainly include

- Degassing from mercury deposits;
- Degassing from aquatic and terrestrial systems (through the reduction of Hg^2+^ to Hg^0^);
- Geological activity—volcanic and geothermal processes (underwater exhalations from geothermal vents);
- Biomass burning—e.g., fires of forests and steppes;
- Erosion of mercury-containing minerals; and
- Plant growth.

Mercury emissions into the air from sources of natural origin are an important element of global mercury flows, as it is the largest element of the global mercury cycle. Many research teams have estimated these emissions, using different numerical models in addition to direct measurements. Their balance is hampered by the share of reemissions from anthropogenic sources which is difficult to estimate (Mason 2009; Pirrone et al. 2010; Pirrone and Cinnirella 2012).

In the aquatic environment, mercury occurs in many forms which depend on the oxidation-reduction conditions. The forms HgCl_4_^2−^ and HgOH^−^ dominate in the good oxidation conditions, whereas sulfur-related forms (HgS^2−^ and CH_3_HgS^−^) prevail in the reduction conditions.

In the intermediate conditions, the alkyl forms of mercury, MeHgCl and EtHgCl, can most often be found (Kabata-Pendias and Mukherjee 2007; Kabata-Pendias 2011; Tyler 1992). Soluble forms of mercury, such as [HgOH]^+^, [HgCl]^+^, [HgCl_2_], [HgCl_3_]^−^, [HgCl]^2−^, and [HgS_2_]^2−^, can often be encountered. Higher concentrations of Cl^−^ ions, which form stable complexes with mercury, such as HgCl_3_^−^, HgCl_2_^−^, HgCl_4_^2−^, or HgBrCl^−^, lead to increased dissolution of solid phases of mercury (Grassia and Nettib 2000).

Moreover, mercury can occur in soluble non-ionic organic compounds and other organic and inorganic compounds. Examples of sparingly soluble forms of mercury include CH_3_Hg^+^ or Hg(CN)_2_. There are many paths of mercury transport into the aquatic environment. Inorganic forms, such as Hg(II) and methylmercury (MeHg), can be directly introduced into reservoirs through (wet and dry) depositions from the atmosphere (Lina et al. 2006). On the other hand, Hg(II) and MeHg are transported into water reservoirs through surface runoff as well as through leaching from the upper levels of a soil profile to groundwater and, subsequently, to surface waters (Fitzgerald et al. 2007; Morel et al. 1998; US EPA 1997).

Wet and dry atmospheric depositions are the most frequent paths of transport of Hg (THg) into the surface waters of the Arctic and the Antarctic. It is estimated that the total annual quantity of mercury penetrating the atmosphere from natural and anthropogenic mercury emission sources is 5000–6500 t (Berg et al. 2006; Bone et al. 2007; Bookman et al. 2008; Camargo 1993; Cheng and Hu 2010; Jiang et al. 2006; Gbor et al. 2007; Gray and Hines 2006; Lamborg et al. 2002; Mason et al. 1994; Streets et al. 2009), with the emissions from anthropogenic sources estimated at about 2200 t Hg/year (Bergan et al. 1999; Gbor et al. 2007; Pacyna et al. 2006). China is the country with the highest mercury emissions (Wang et al. 2016). Every year, thousands of tons of mercury from air deposition are transported into aquatic ecosystems. Mercury-containing flowing and standing surface waters, such as rivers, streams, and estuaries, are the main paths of mercury transport into marine ecosystems.

In the aquatic environment, mercury undergoes many different chemical and biochemical processes which condition its speciation and transport between the solid and aqueous phases (Fitzgerald et al. 2007; Morel et al. 1998). In the aquatic environment (water, sediments, aquatic fauna, and flora), most mercury occurs in organic and inorganic forms of divalent mercury and Hg(0), as a form of mercury dissolved in the aqueous phase (Ullrich et al. 2001). Mercury adsorption and desorption processes in the aquatic environment play a dominating role in the distribution of different forms of mercury in the particular elements in the aquatic environment. In waters, these processes are also responsible for the course of mercury transport, transformation, and uptake by living organisms, and they also condition the toxicity of this element.

In the aquatic environment, mercury can be adsorbed on sediment particles, thus constituting a substantial mercury resource. In the sediments in water reservoirs, both as a result of chemical reactions and under the impact of biological factors, e.g., those related to the activity of microorganisms, methylmercury, CH3Hg^+^, and dimethylmercury, (CH_3_)_2_Hg, emerge (Benoit et al. 2003; St. Louis et al. 1994).

MeHg is the most common form of organic mercury in the environment. Methylmercury is a neurodevelopmental toxicant (Obi et al. 2015), and it is also the most toxic form of mercury (Henriques et al. 2015). MeHg and dioxin-like compounds are considered as the most important toxic compounds in the case of large-scale consumers of seafood (Szefer 2013).

Methylation is a result of abiotic and biotic processes, which are affected by such factors as pH, temperature, the presence of sulfates, and the availability of organic carbon.

The impact of mercury in the environment on human health was found for the first time in relation to the Minamata disease in the 1950s, which caused mass-scale poisoning by methylmercury. It had accumulated in aquatic organisms which were subsequently eaten by humans. A similar case of poisoning by mercury accumulated in fish also took place in Sweden (Zaib et al. 2015).

Apart from spectacular cases of poisoning, the presence of mercury in the environment also affects the human population in a more concealed manner. Every year, Trasande et al. (Trasande et al. 2005) found mercury concentrations exceeding 5.8 μg/L—a level related to IQ loss—in blood samples taken from 316,588–637,233 children. Humans are mainly exposed to methylmercury as a result of their consumption of oceanic fish (Drevnick et al. 2015).

One of the key stages of the biogeochemical cycling of mercury in the environment is its biomobilization, which is mediated by microorganisms. Microorganisms mediate in the following four types of mercury transformation: in the reduction of Hg(II) to Hg(0), in the degradation of CH_3_Hg(I) and other organic mercury compounds, in the methylation of Hg(II), and in the oxidation of Hg(0) to Hg(II). The transformation processes listed above also unfold in the environment without any involvement of microorganisms. Table 1 shows a summary list of known mercury transformation mechanisms.

**Table 1:** Mechanisms of mercury transformation processes

Mechanisms	Reduction of Hg(II)	Degradation/demethylation of CH_3_Hg(I)	Methylation	Oxidation of Hg(0)
Biotically	Enzymatically—mercury reductase	Enzymatically—organomercurial lyase (OL)	Transfer of methyl groups by corrinoid coenzyme (bacterial methyltransferase)	Hydroperoxidase (e.g., catalase)
	Indirectly—reduced metabolites	Oxidative demethylation—anaerobic bacteria	Disturbances in methionine synthesis pathway in fungi	–
Abiotically	Free radicals related to humic substances	Photodegradation	Photochemically—induced by humic and fulvic acids	In the atmosphere via H_2_O_2_ under low pH conditions
	Disproportionation of Hg(I)	–	Transformation of CH_3_Hg(I) to (CH_3_)Hg in the presence of H_2_S	–

Source: Barkay (2000)

Biological mercury transformations, along with the processes of its chemical transformations, provide the basis for mercury cycling in the biosphere; furthermore, the role played by the biological transformations themselves is not fully known and probably depends on mercury concentrations and the conditions occurring in the environment.

The reduction of Hg^2+^ and the degradation of organic mercury compounds are processes constituting natural detoxification mechanisms, which unfold in bacteria and enable their growth and development in the presence of these toxic compounds. Bacteria that are resilient to the harmful impact of Hg^+2^ are capable of producing mercury, which catalyses the reaction with the involvement of nicotinamide adenine dinucleotide phosphate (NADPH). Due to its low water solubility and high vapor pressure, Hg^0^ which emerges as a result quickly escapes into the atmosphere. Some bacteria which are resilient to Hg^2+^ due to the presence of organomercurial lyase (OL) also demonstrate resistance to the toxic impact of organic mercury compounds (Walsh et al. 1988). The resistance to the harmful impact of organic forms of mercury is related to the action of both enzymes, lyase and reductase, while all the bacteria which are not vulnerable to the impact of organic mercury compounds are at the same time resilient to the toxic effect of Hg^+2^ (a wide range of resilience). However, a large part of bacteria do not show the capacity to produce organomercurial lyase; as a result, they are vulnerable to the presence of this form of mercury in the environment (a narrow range of resilience).

Whereas the reactions of the reduction of Hg^2+^ to Hg^0^ may unfold without any involvement of microorganisms, in contrast, the process of the degradation of organic forms of mercury without any involvement of microorganisms unfolds extremely slowly and is of no greater significance in the processes of mercury cycling in nature (Barkay et al. 1992). The chemical reactions in which Hg^0^ escapes into the atmosphere include the reduction of Hg^2+^ to 2Hg^+^, with the subsequent disproportionation to Hg^0^ and Hg^2+^ (Baltisberger et al. 1979), and the reduction through the interaction of Hg^2+^ with free-radical electrons of humic acids (Alberts et al. 1974).

As shown in Table 1, abiotic sources of methylmercury include transformations of Hg^2+^ as a result of its reactions with fulvic and humic acids (Nagase 1984) and the transfer of methyl groups from the soluble fraction of organic matter to Hg^2+^. These reactions are initiated phototochemically in the presence of sulfur (Akagi et al. 1974).

The intensity of methylation grows as the temperature and the content of Hg(II) in the environment increase, while the optimum level is reached at pH of less than 5.

The methylation reaction is also stimulated by the presence of other metals which play the role of catalysts. Photochemical methylation takes place in the presence of chemical donors of methyl groups, such as methanol or ethanol; moreover, the greatest efficiency is achieved in the presence of acetic acid. The anaerobic conditions and the presence of colored compounds as photosensitizers also enhance the efficiency of this process (Barkay 2000).

## Mercury in Marine and Oceanic Waters

In the biogeochemical mercury cycling, a substantial part of it reaches seas and oceans and its important source is its atmospheric deposition (Bindler 2003; Wang et al. 2004).

In oceanic waters, mercury mainly occurs in the forms of Hg^0^, Hg^2+^, MeHg, and diMeHg and in colloidal form (Morel et al. 1998). In marine waters, mercury forms compounds with chlorine (HgCl_3_^−^ and HgCl_4_^2−^) to a greater extent than oxides, as is the case in freshwaters (Mason and Fitzgerald 1993). It has been demonstrated that in saltwaters, Hg^2+^ forms complexes with halides, such as those of chlorine, and these complexes do not undergo the reduction and methylation processes (Gardfeldt et al. 2003; Whalin et al. 2007) as quickly as the other Hg^2+^ compounds do. Dissolved gaseous mercury (DGM) arises as a result of the transformations of both Hg^0^ and Me_2_Hg (Lamborg et al. 1999; Mason et al. 1995), with the form Hg^0^ dominating in the upper layer of the ocean (Table 2) (Gardfeldt et al. 2003; Laurier et al. 2003).

**Table 2:** Mercury concentrations in marine waters in the different parts of the world, in ng/L

Survey site	THg^a^	HgCR^a^	DGM^a^	diMeHg^a^	HgR^a^	MeHg^a^	HgC^a^	Source
	Seawaters							
Minamata Bay (Japan)	1,600–3,600	–	–	–	–	–	–	Hosohara et al. (1961)
Atlantic Ocean	400–1,600	–	–	–	–	–	–	Aidin’yan and Belavskaya (1963)
Red Sea	700–2,000	–	–	–	–	–	–	Aidin’yan (1962)
Pacific Ocean, Ramapo Deep	80–150	–	–	–	–	–	–	Hamaguchi et al. (1961)
Baltic Sea	0.6 ± 0.2	–	–	–	–	∼20 % THg	–	Pempkowiak et al. (1998)
Gulf of Gdańsk	277–630	–	–	–	–	–	–	BMEPC (1987)
Gulf of Finland	10–140	–	–	–	–	–	–	
Bothnian Bay	2–40	–	–	–	–	–	–	
Gulf of Riga	10–40	–	–	–	–	–	–	
Matsalu Bay	5–130	–	–	–	–	–	–	
North Sea	0.56 ± 0.24	–	–	–	–	–	–	Coquery and Cossa (1995)
North Sea	0.5–200	–	–	–	–	–	–	Schmidt (1991)
Mediterranean Sea (Tunisia)	990–27,060	–	–	–	–	–	–	Nasfi (1995)
Atlantic Ocean (N–E)	2.7	–	–	–	–	–	–	Cossad et al. (1996)
Sepetiba Bay (Brazil)	–	0.12–0.67	0.032–0.092	–	0.11–0.36	<0.02–0.55	–	Marins et al. (2000)
Sepetiba Bay (Brazil)	–	–	<2.6	–	0.2–32.2	0–246	–	Marins et al. (2000)
South China Sea	0.8–2.3	–	–	–	–	0.05–0.22	–	Fu et al. (2010)
Andaman Sea (Thailand)	<10–540	–	–	–	–	–	–	Thongraa-Ra and Parkpian (2002)
Yellow Sea	1.34–5.5	–	–	–	–	–	–	Ci et al. (2011)
Wuli Estuary	39–430	–	–	–	–	0.046–0.28	–	Wang et al. (2009a)
Pacific Ocean (N)	–	–	–	–	–	173 ± 118^b^	–	Sunderland et al. (2009)
Pacific Ocean (equatorial part)	–	–	–	<10–670^b^	–	<50–500^b^	–	Mason and Fitzgerald (1990, 1993)
Atlantic Ocean (N)	–	–	–	81 ± 68^b^	–	ND^c^	–	Mason et al. 1998
Atlantic Ocean (S and equatorial part)	–	–	–	<10–110^b^	–	25–200^b^	–	Mason and Sullivan (1999)
Mediterranean Sea	–	–	–	3 ± 3^b^	–	280 ± 120^b^	–	Kotnik (2007)
	–	–	–	3 ± 2^b^	–	290 ± 26^b^	–	Horvat et al. (2003)
	–	–	–	<20–290^b^	–	<150^b^	–	Cossa et al. (1997)

*–* no data or not applicable

^a^ *THg* total Hg, *HgCR* total dissolved Hg, *DGM* dissolved gaseous Hg, *HgR* reactive Hg, *diMeHg* dimethylmercury, *MeHg* monomethylmercury, *HgC* Hg in elemental form

^b^Value given in fM

^c^ND below the detection limit

The impact of anthropogenic sources on the contents of mercury and its forms can be seen to a greater extent in the surface layers of the oceans (i.e., in the upper layer of the ocean waters down to a depth of about of 100 m) (Strode et al. 2007). In the near-surface layer, i.e., in the layer between 100 and 1000 m, which is the water-mixing layer, the displacement of mercury of anthropogenic origin is hampered. It depends on many factors, e.g., the local and regional movements of water masses, on the production formation and degradation, as well as the accumulation of intermediate substances (Mason and Sheu 2002).

The particular oceans are different in their total mercury concentrations, with the average concentration of about 1.5 picomoles (pM) (Lamborg et al. 2002). Higher concentrations were recorded in the Mediterranean Sea—2.5 pM (Cossa et al. 1997), and the northern part of the Atlantic—2.0 pM (Mason et al. 1998), whereas a lower concentration was found in the Antarctic Ocean—0.8 pM (Laurier et al. 2004; Sunderland and Mason 2007). Simulations carried out using mathematical models have indicated that the mercury concentrations in most oceans are not balanced with its atmospheric deposit and that they will also systematically grow over the next several dozen years (Sunderland and Mason 2007).

The mercury exchange with the ocean water currents unfolds most quickly in the Atlantic Ocean (17.99–24 Sv), while in the other intervals, it has similar values, falling within the range of 6 to 12.4 Sv.

It is believed that the mercury exchange at the interface between the ocean surface and the atmosphere unfolds relatively quickly. Just as in the freshwater systems, Hg transformation processes in saltwaters are quite complex, too. Hg^2+^ reaches the ocean through its dry and wet depositions, while Hg^0^ does so through its dry deposition; in addition, Hg2+ subsequently undergoes biological or photochemical reduction to Hg^0^ and absorption on the solid particles of the suspended sediment or methylation (Strode et al. 2007).

It is difficult to determine the intensity of the methylation process in saltwaters, since old data are not reliable given the analytical detection limits of measurement equipment; in consequence, difficulties emerge in respect of the MeHg measurements in these waters. The average levels of MeHg contents determined in oceanic waters fell in the range of 2–35 % (Sunderland et al. 2009). In saltwater systems, mercury methylation may unfold in sediments in the continental shelf areas (Hammerschmidt and Fitzgerald 2006), in estuaries (Gill et al. 1999; Heyes et al. 2006), in a seawater column (Monperrus et al. 2007), or in hydrothermal vents situated in deep ocean layers (Kraepiel et al. 2003).

In the relevant literature, the following two models describing the fate and behavior of mercury in saltwater reservoirs have been presented: a conceptual model which treats all the oceans as one ocean, based on the data and modeling presented by Rolfus and Fitzgerald (1995), and the “ocean margin” model developed by Cossa et al. (1996), providing that the ocean margins manifested themselves as the convergence of continents and oceans, covering such geological features, such as estuaries, inland seas, and the continental shelf. However, it should be borne in mind that these models are very general and only reflect basic knowledge of the mercury paths and transformations in the marine environment.

### The Fate of Mercury in Saltwaters (a Conceptual Model of One Ocean)

Rolfus and Fitzgerald (1995) created a simple ocean model, designed to help determine the fate of mercury in saltwaters and its accumulation in fish and mostly based on the data which these authors had collected earlier. The model provided that the ocean consisted of the following three compartments: the coastal zone, the upwelling zone, and the open ocean. The open ocean zone represents more than 90 % of the total surface area of the oceans, but it is characterized by low fish productivity. The coastal and upwelling zones represent, respectively, about 10 and 0.1 % of the total surface area and generate almost all the fish production (in the model, each such area accounts for almost 50 % of the total fish production). The mercury sources adopted in the model include atmospheric deposition from the atmosphere, the flux from river systems, and the flux from deep ocean waters. On the basis of the dependence observed in the North Atlantic between the increased concentration of reactive mercury in the surface ocean waters and the predicted rate of atmospheric deposition, it is also assumed that its point of entry is the mixed layer of the waters. From this layer, reactive mercury is transported in the form of complexes with suspended particles to the regions and layers of the ocean where its methylation occurs (these areas are naturally poorer in oxygen) and mercury is released from the complexes as they fall into lower regions of the reservoir. This model also assumes that monoMeHg forms in regions which are poor in oxygen, under the thermocline of the open ocean zone and the upwelling zone, from where it is subsequently transported to the mixed layer, to a depth of less than 100 m, where it is incorporated into the lower levels of the trophic chain (Mason and Fitzgerald 1990, 1993, 1996). After its transport from the mixed layer, in the subthermocline water layer, most of reactive mercury is methylated to dimethylmercury (diMeHg), although monomethylmercury can also form directly from reactive mercury. In marine waters, dimethylmercury is unstable and most of it quickly decomposes to form MeHg (Mason and Fitzgerald 1996). Some of MeHg is then converted to Hg^0^, which is transported to the surface zones of the ocean. In this layer, as a result of its supersaturation, elemental Hg can be released into the atmosphere. This is the main mechanism of removal of mercury from saltwaters, reaching a level of 1 % per day in the open ocean zone. It is presumed that the transport of reactive mercury from the mixed layer of the ocean is a factor which determines the rate of methylmercury formation (Mason and Fitzgerald 1996).

The reduction processes are related to both abiotic and biotic factors of the marine environment (Mason et al. 1995); specifically, abiotic factors are responsible for about 10–30 % of the reduction. It was demonstrated that the abiotic reduction might unfold under the impact of sunlight. In an experimental aquatic system, they demonstrated that in the conditions of the combination of fulvic and humic acids with a synthetic light source, the reduction of dissolved divalent forms of Hg occurred. In turn, Weber (1993) suggested that the process of abiotic mercury reduction was mediated by methyltin compounds (e.g., MeSnCl_3_, Me_2_SnCl_2_, Me_3_SnCl) and humic acids. However, for the most part, the reduction of reactive mercury results from the action of microorganisms, e.g., bacteria and cyanobacteria in the mixed layer of the ocean (Mason et al. 1995).

The model described here also assumed that mercury methylation in the coastal compartments (including the estuary regions) occurred in both the sediments and in the water column, in the vicinity of the oxycline (Rolfus and Fitzgerald 1995), from where methylmercury was transported to the mixed layer and incorporated into the lower trophic levels of the marine food web. The total deposition of mercury to marine reservoirs was estimated at 10 Mmol in a year, while the input from rivers and their estuaries was estimated at about 10 % of this value (about 1 Mmol/year); in turn, the input to the upwelling layer from deeper and colder waters was estimated at about 0.5 Mmol/year. Several authors, including Rolfus and Fitzgerald (1995), found that the enhanced mercury deposition resulting from higher anthropogenic emissions caused greater bioaccumulation in the food chain and, primarily, higher mercury contents in marine fish.

### The Fate of Mercury in Saltwaters (the *Ocean Margin* Model)

Estuaries and coastal regions are particularly vulnerable to anthropogenic mercury contamination, since contaminants are both transported to them in river waters and discharged directly to the oceanic waters. In these regions, there is a higher level of reactive forms of mercury and particle-bound mercury released into the atmosphere from local sources of anthropogenic origin. It is well known that reactive Hg deposits faster than elemental Hg and that higher concentrations of oxidants may occur in the atmospheric layer over the coastal waters. On the basis of research results, it has been found that in certain mercury-contaminated estuaries, the main source of their contamination is the direct discharge of mercury into water rather than its atmospheric deposition. On the basis of data collected for the regions mentioned above, Cossa et al. (1996) developed a mass balance model for mercury for these areas and the quantity of THg estimated on the basis of the model was about 3.3 Mmol. In this model, it was assumed that the mercury flux from the river systems to the oceanic margins represented the highest share of the total mercury input to the coastal waters and that annually, about 4.8 Mmol of mercury reached oceanic waters in this way. Cossa et al. (1996) also noted that the concentrations of total mercury were very variable and that, moreover, the highest concentration was found in the waters of rivers flowing through urbanized and industrial areas. More than 90 % of mercury are transported in particle-bound form. Moreover, a substantial part of this form of mercury seems to be unreactive and is deposited in sediments. The authors estimated that the dry deposition of mercury in coastal waters amounted to about 2 Mmol/year and found that a substantial part of mercury was chemically reactive and participated in reactions unfolding in the marine environment.

Atmospheric deposition in remote ocean areas and then its transport via upwelling to these regions (2.5–3.5 Mmol/year) were also considered to be a significant source of mercury in the waters of marine coastal regions.

The impact of anthropogenic sources on the contents of mercury and its forms is more conspicuous in the surface layer of the oceans (i.e., in the upper layer of the ocean waters down to a depth of about of 100 m) (Strode et al. 2007). In the near-surface layer, i.e., in the layer between 100 and 1000 m, which is the water-mixing layer, the displacement of mercury of anthropogenic origin is hampered. It depends on many factors, e.g., the local and regional movements of water masses, on the formation and degradation, and the accumulation of intermediate substances (Mason and Sheu 2002).

It is believed that the process of mercury exchange at the interface between the ocean surface and the atmosphere unfolds relatively quickly. Just as in freshwater systems, Hg transformation processes in saltwaters are also quite complex. Hg^2+^ reaches the ocean through its dry and wet depositions, while Hg^0^ does so through its dry deposition; in addition, Hg^2+^ subsequently undergoes biological or photochemical reduction to Hg^0^ and absorption on the solid particles of the suspended sediment or methylation (Strode et al. 2007).

It is difficult to determine the intensity of the methylation process in saltwaters, since old data are not reliable given the analytical detection limits of measurement equipment; in consequence, difficulties emerge in respect of the MeHg measurements in these waters. The average levels of MeHg contents determined in oceanic waters fell in the range of 2–35 % (Sunderland et al. 2009).

In saltwater systems, mercury methylation may unfold in sediments in the continental shelf areas (Hammerschmidt and Fitzgerald 2006), in estuaries (Gill et al. 1999; Heyes et al. 2006), in a seawater column (Monperrus et al. 2007), or in hydrothermal vents situated in deep ocean layers (Kraepiel et al. 2003).

The mercury flux from sediments to the waters in coastal waters was regarded as its less significant source.

Taking into account the assumptions listed above and enumerating them in approximate order of significance, it is found that the flux of total mercury in the area of coastal waters includes, *inter alia*,

- The sedimentation of particles, with mercury adsorbed on their surface originating from surface flow in riverine and deep regions;
- The transport from the open ocean waters; and
- The emissions into the atmosphere which are, on a global scale, usually offset by atmospheric deposition, with a regional imbalance resulting from the domination of deposition processes over releases in the northern latitudes and a converse situation in the southern latitudes.

The model in question was also used to describe the fate and behavior of methylmercury in oceanic coastal waters (Cossa et al. 1996). It follows from the assumptions of the model that more than half of MeHg present in oceanic coastal waters originates from the waters of the bottom of the ocean, whereas the other part consists of the product of methylation of reactive mercury in coastal waters and MeHg from other sources. Mercuric ions in the anoxic layers of sediments in marine water reservoirs are transformed to monomethylmercury, primarily through microbial transformations driven by sulfate-reducing bacteria, such as *Desulfovibrio desulfuricans* (Compeau and Bartha 1985), and the organic matter content has a significant impact on the course of the process (Choi and Bartha 1994). Research has demonstrated that the bioavailability of MeHg bound to sediment particles is substantially greater than the bioavailability of divalent inorganic mercury bound to sediment particles. Dissolved species of both methylmercury and divalent inorganic mercury demonstrate greater bioavailability than particle-bound forms (Choi and Bartha 1994).

## Mercury in Benthic Sediments

Both in soil and benthic sediments, mercury is mainly related to the presence of organic matter. In sediments, methylmercury which has formed through methylation represents not more than 1.5 % of the total quantity of mercury; this quantity is determined as an equilibrium level between the formation and removal processes. In each aquatic environment, a certain state of equilibrium is established between the reactions of methylation and demethylation, depending on the stability of the particular forms of mercury. Dimethylmercury is unstable in sediments but retains relatively high stability under the impact of such factors as its high sulfate content, salinity, anaerobic conditions, or continuous access to methane (Weber et al. 1998).

When comparing the results of the measurements of mercury contents in samples taken from sediments, it should be taken into consideration that slight changes in the conditions of the storage of the collected samples (temperature, redox conditions, and oxygenation) have a very large effect on changes in the contents of the particular forms of mercury (Horvat and Gibicar 2005).

The mercury content in benthic sediments usually reflects its level in the water reservoir, particularly in reservoirs situated in contaminated areas (mines, metallurgical plants, chemically protected crops). Table 3 shows the concentrations of mercury and its compounds determined in the sediments in surface waters in the different parts of the world. There are substantial differences in mercury concentrations among the sediments originating from different water reservoirs and different regions (Table 3).

**Table 3:** Mercury concentrations in marine sediments, in mg/kg d.s.

Location	HgT total mercury	MeHg	Source
Marine sediments			
Baltic Sea	2–340	–	Pempkowiak et al. (1998)
Baltic Sea Proper	100 ± 50	–	Borg and Jonsson (1996)
Baltic Sea (Aland Sea)	180 ± 60	–	
Baltic Sea (Bothnian Sea)	100 ± 30	–	
Bothnian Bay	400 ± 240	–	
Gulf of Puck	0.74–5.7	–	Falandysz et al. (1993)
Gulf of Gdańsk	3.5–160	–	
Gulf of Gdańsk	0.25	–	Szumiło-Pilarska et al. (2016)
Gulf of Puck	2.8–180	–	Boszke et al. (2002)
Denmark Strait	60–220	–	Brzezińska et al. (1984)
Baltic Sea (Bosex Area)	140–190	–	
South Baltic Sea	30 ± 10	–	
Baltic Sea Proper	20–360	–	
Gulf of Gdańsk	310 ± 310	–	
Gulf of Riga	30–790	–	Ojaver (1995)
Mediterranean Sea (Israel)	10–900	–	Herut et al. (1994)
Mediterranean Sea (Italy)	100–5,330	–	Barghigiani and Ristori (1995)
South China Sea (Malaysia)	20–127	0.01–0.053	Kannan and Falandysz (1998)
Victoria Harbour, Hong Kong	47–855	0.1–1.5	Shi et al. (2007)
East China Sea (China)	<0.0005–0.0798	–	Shi et al. (2005)
	0.0041–0.0476	–	Fang and Chen (2010)
	0.042–0.072	–	Fang et al. (2004)
Coastal sediments			
Bay of Fundy, (USA/Canada)	25–514^a^	0.5–7.38	Sunderland et al. (2006)
Hainan Coast	0.02–0.1	–	Qiu et al. (2011)
Southeast China Coast	0.0023–0.9036	–	Ding et al. (2009)
Andaman Sea (Thailand)	0.047–2.135	–	Thongraa-Ra and Parkpian (2002)
Bohai Sea	0.8–25	–	Wang et al. (2009b)

^a^pmol/g d.s.

It is believed that natural THg concentrations fall within the interval from 10 to 200 ng/g d.s. (Fergusson 1990), while the quotient of the THg concentration and methylmercury can be used as a marker to describe the state of contamination of a reservoir (Kannan and Falandysz 1998); moreover, in the uncontaminated regions, this indicator takes values of less than 1. In the benthic sediments of the Gulf of Gdańsk, the determined average concentration of methylmercury was 0.65 ng/g d.s., representing about 0.5 % THg, while the indicator in question fell within the interval from 0.02 to 2.27. The direction of mercury transformations in the anoxic conditions of the lower layers of the water column and benthic sediments is determined by a high affinity of mercuric ions with sulfides.

In the anoxic conditions, the dominant forms of mercury include HgS, HgS_2_H_2_, HgS_2_H^−^, and HgS_2_^2−^, as well as CH_3_HgS (the most important form of mercury in these conditions). Mercuric sulfide HgS, which is sparingly soluble in the aquatic environment, is mainly deposited in benthic sediments and determines the solubility of Hg(II) in waters with low oxygen content or without oxygen; moreover, it is believed that the deposition of HgS in sediments is correlated with the contents of organic matter and iron oxide ions (Boszke et al. 2002). The solubility of HgS increases with the growing content of sulfide ions, which enable the formation of soluble complexes with mercury. This is of large importance in the anoxic areas of aquatic ecosystems, where there are high concentrations of dissolved mercury. Although mercuric sulfide HgS is strongly bound to sediments, part of it may occur in dissolved form as a result of microbial transformations or under the impact of the aerobic conditions (Gagnon et al. 1997). The redeposition of mercury from benthic sediments to the water column occurs as a result of diffusion or the activity of benthic organisms. Moreover, in the latter case, it is greater by a factor of 2 to 10 than the redeposition as a result of diffusion (Rutgers van der Loeff et al. 1984). It is also believed that the existence of oxidizing conditions in the surface layer of sediments constitutes a geochemical barrier to the diffusion of methylmercury from the near-surface layers of sediments with oxidizing conditions to the water column. The mercury input from deeper sediment layers to the near-surface layers of sediments as a result of diffusion was estimated at 3 % of the total mercury input (Gagnon et al. 1997).

Research carried out in the water-sediment system demonstrated that Hg(II) underwent strong sorption on clay particles and biotic elements, which was much weaker than that on sand particles. It was demonstrated that it also underwent strong complexation on organic matter. Following their absorption on solid elements, Hg(II) and methylmercury settle and accumulate on the surface of the sediment layer, which undergoes dynamic transformations on the boundary of the water column, resuspension and bioturbation. The content of methylmercury in water and sediments is a function of its transformations in the methylation and demethylation processes. In the aquatic environment, methylmercury undergoes degradation under the impact of two microbial transformations and under the impact of light (Barkay et al. 2003).

Equilibrium research demonstrated that the in situ formation of mercury was often its main source in water reservoirs and benthic sediments (Benoit et al. 2003; Gilmour et al. 1998). Despite the contamination of aquatic ecosystems with mercury from anthropogenic sources, its direct increase can be observed in aquatic organisms rather than in these ecosystems. This primarily results from the presence of regulatory mechanisms, such as the activity of methylating microorganisms and the contents of organic matter and sulfur (Grieb et al. 1990). The quantity of accumulated mercury in aquatic organisms is affected by such factors as the level in the trophic chain (the higher level in the trophic chain it is, the more mercury is stored in it), the age of organisms, the microbial activity, the mercury concentration in the sediment layer, the content of the soluble fraction of organic matter, salinity, pH, and the redox potential (Kamman et al. 2005). The benthic sediments of oceans are regarded as the ultimate “recipient,” which cumulates highly insoluble forms of HgS (Whalin et al. 2007; Wiener et al. 2003). The first stage related to mercury bioaccumulation in aquatic ecosystems is the transformation from its inorganic form to methylmercury, which occurs in both the aquatic and sedimentation phases. Although the mercury bioaccumulation process prevail over its demethylation, the mechanism of methylmercury synthesis has not been completely identified (Horvat 1996).

Cores of sediments from distant, uncontaminated areas are used to study mercury deposition in historically remote times. The overall background THg level demonstrated by Fuji (1976) is about 50 μg/kg in river sediments, 100–300 μg/kg in lake sediments, and 50–80 μg/kg in marine sediments. The MeHg content in the pool of total mercury (THg) reaches about 10 % (US EPA 1997).

Mercury is a strongly dispersed element. Its contents in sedimentary and magmatic rocks are similar, i.e., about 10 μg/kg. Higher mercury contents can be found in rocks which are rich in organic matter, i.e., in clay rocks. Natural mercury contents in sediments do not exceed 0.05 mg/kg. Due to the poor solubility of mercuric carbonates, phosphates, and sulfides and also due to the formation of bonds and connections with organic compounds, mercury undergoes ionic adsorption by organic and inorganic substances. Mercury adsorption is conditioned by many factors, depending, e.g., on the chemical form of mercury, the quantities and chemical properties of organic and inorganic colloids in sediments, the type of cations in sorption complexes, and the redox potential or the reaction of sediments. An increase in the mercury content in surface sediments is usually related to human activities in a given area, but it is rarely affected by geological factors, such as the presence of underlying mercury-containing rocks. The major mercury sources in sediments are the same as the mercury sources in soils (e.g., atmospheric deposition, pesticides, the application of sewage sludge and municipal waste for soil fertilization, coal combustion, metallurgy, and so-called artisanal and small-scale gold mining (ASGM)) and surface waters from which Hg migrates to sediments (Lacerda and Salomons 1999).

## Mercury Bioaccumulation in Aquatic Organisms

As a result of the intensive fixation of mercury by bottom sediments, there is the risk of its accumulation in aquatic organisms (Wilken and Hintelmann 1991). Surveys on different species of seawater and freshwater fish have indicated that mercury concentrations in their tissues grow as their body mass and age increase (Squadrone et al. 2013).

The cycle of mercury transformations in the aqueous environment is a complex one. Biomethylation, the products of which can easily move between the different elements of the environment, may play an important role in the transfer of mercury. Inorganic compounds of mercury (II) in the aqueous environment undergo biochemical transformations effected by microorganisms in the anaerobic conditions, as a result of which highly toxic methylmercury compounds emerge. Both metallic mercury and the mercury contained in different compounds undergo biomethylation in hardly soluble sediments on the bottom of water reservoirs (Boening 2000; He et al. 2005; Squadrone et al. 2013; Stein et al. 1996; Wang et al. 2004; Winfrey and Rudd 1990).

It is important to investigate the methylmercury absorption and accumulation pathways in the food chain. For this purpose, research is carried out on mercury concentrations in animal tissue and determines the position which these organisms occupy in the food chain—by using the stable nitrogen isotope ^15^N (the content of which in tissues grows with each successive trophic level occupied by the consumer) (Post 2002; Roach et al. 2013). Short-chain alkyl mercury compounds, including those of metylmercury, demonstrate strong bioaccumulation properties. As a lithophilous compound, methylmercury is also capable of easily penetrating through mucous membranes.

A percentage share of mercury in the form of methylmercury in organisms grows for a higher trophic level. Mostly because of their small body size, short lifetime, and low position in the food chain, aquatic invertebrates accumulate smaller amounts of methylmercury in their organisms compared with those in fish. Wong et al. (1997) demonstrated that an average mercury concentration in invertebrates fell within the range from 0.07 to 0.18 μg/g. Surveys to determine the mercury contents in aquatic organisms have also been carried out on crayfish. They are considered to be scavengers which feed on the lowest trophic levels; as a result of this, the methylmercury contents in their bodies are slow compared with its contents in organisms situated at higher trophic levels.

The pollution caused by mercury is a serious threat for the marine environment. This is both a hygienic and ecotoxic problem. When this element occurs at levels exceeding its natural level in the seas and oceans, it poses a large threat for aquatic organisms.

Mercury concentrations detected in fish tissues vary, depending, e.g., on the fish species or the sea area from which a given fish originates. This is important when consumers choose a product. It is important to compare the place of origin of the fish contained in a fish product with the list of sea areas which are generally recognized to be highly vulnerable to mercury pollution.

Mercury contents in the organisms of sea fish depend not only on their species. The surveys carried out by Monteiro et al. (1996) on eight North Atlantic fish species demonstrated that mercury concentrations in the fish varied depending on the depth where they lived. The average mercury concentrations in the fish species covered by the surveys varied between 57 and 377 ppb in fresh tissue and were four times higher in mesopelagic species (depth of more than 300 m) than those in epipelagic (depth of less than 200 m). The fate of organic Hg species in marine ecosystems is dependent also upon surface water temperature, nutrient supply, and on the abundance of phytoplankton and its species composition (Szefer 2002).

In addition to the depth where sea fish feed, the mercury contents in their bodies are also affected by the fact whether they live in open waters or those situated closer to the coast. The coastal regions of seas and oceans are particularly vulnerable to anthropogenic mercury contamination, since both contaminants from rivers and those discharged directly into the oceans are transported to those areas. An additional source of mercury in these sea areas is the mercury exchange at the interface between the ocean surface and the atmosphere. Table 4 lists the data on mercury contents in fish coming from different sea areas in the world. These data demonstrate a large scatter of mercury contents both between the fish species and the water areas.

**Table 4:** Mercury contents in various sea fish from different sea areas in the world

Origin of sample	Concentration	Unit	Source
Caspian Sea	<0.05–0.79	mg/kg DM	Anan et al. (2005)
Pacific Ocean, Alaska	0.19–0.40	mg/kg DM	Meador et al. (2005)
Pacific Ocean, California	0.24–0.73	mg/kg DM	
Barents Sea, Greenland	0.19–1.10	mg/kg FM	Julshamn et al. (2006)
Indian Ocean, Mozambique	0.21–3.97	mg/kg DM	Kojadinovic et al. (2007)
Atlantic Ocean, Ghana	0.004–0.122	mg/kg FM	Voegborlo and Akagi (2007)
Atlantic Ocean, Azores	0.19–1.44	mg/kg FM	Afonso et al. (2007)
Black Sea, Turkey	0.025–0.084	mg/kg FM	Tuzen (2009)
Baltic Sea, Poland	0.018–0.118	mg/kg FM	Polak-Juszczak (2009)
Adriatic Sea, Croatia	0.001–0.52	mg/kg FM	Bilandžić et al. (2011)

*DM* dry mass, *FM* fresh mass

## Mathematical Models

The management strategies intended to control the emissions of mercury of anthropogenic origin require the identification of the response of ecosystems to changes in the atmospheric deposition of this element. Mathematical models are valuable tools which provide information about the possible responses of ecosystems, since the measured data themselves are often insufficient and cannot be extrapolated in order to investigate the environmental impacts in other systems (Knightes et al. 2009).

Environmental models are an important decision-making tool for authorities. The spatial and temporal scales linking the tasks related to the control of the environment and the protection of its quality usually do not enable a comprehensive approach to the problem, and thus, they do not make it possible to understand the relation between economic activity and environmental quality. Moreover, environmental models facilitate the understanding of the key research needs and priorities of the data collection in the future (NRC 2007).

There are many models which are developed to simulate mercury emissions, both at a small scale, for local water reservoirs, and at a global scale, as well as to model mercury bioaccumulation in the chain web of aquatic systems. These models are used, e.g., to investigate the time scale required for the bioaccumulation of a specific mercury content level in the tissues of predators, in response to the differentiated sources of this element in the reservoir. These models are based on data on the mercury concentrations in entire aquatic ecosystems for which simulations are carried out and in the hydrodynamic processes (surface runoff, seepage, leaching, evaporation/evapotranspiration, etc.).

As the appropriate model is designed and developed, its important component are the data on which the model will be based, in particular the data on the dynamics of the processes unfolding in sediments, in the water column, and at the interface between them (Knightes et al. 2009). Table 5 shows the selected parameters used to develop the mathematical models and programs for mercury cycling in the environment. Table 6 presents a summary comparison of certain of the available models used to simulate the fate and behavior of mercury in the aquatic environment.

**Table 5:** The parameters used in the models of mercury recycling in the environment

Chemical properties	Elemental Hg		Inorganic Hg^2+^		MeHg^+^	
Air/water partition coefficient *K*_AW_	0.117	Estimated by Clapeyron for Henry’s law constant at 25 °C	NA^a^	–	9.00 × 10^−6c^	Recommended literature value
Fraction absorbed on the skin	NR^b^	–	0	EA (2009)	0.1	EA (2009)
Diffusion coefficient in the air, m^2^/s	6.34 × 10^−6^	Estimated by the method according to Heinsohn and Cimbala (2003)	NA	–	8.61 × 10^−6^	Estimated by the FSG method
Diffusion coefficient in water, m^2^/s	2 × 10^−9^	Estimated by the method according to Hayduk and Laudie (1974)	NA	–	1.7	Recommended literature value
log Kow—octanol/water partition coefficient (log), dimensionless	0.62	Recommended literature value	NA	–	1.9	Estimated using a non-hydrophobic relation with the octanol/water partition coefficient log Kow
log Koc/w—organic carbon/water partition coefficient (log), cm^3^/g	4.16^d^	–	NA	–	251.1	Recommended literature value
Relative molar mass, g/mol	200.59	Recommended literature value	NA	–	NA	–
Soil/water partition coefficient, cm^3^/g	NA	–	500	Recommended literature value	1.13 (25 °C)	Recommended literature value
Vapor pressure, Pa	0.07028	Recommended literature value	NA	–	100 (21 °C)	Recommended literature value
Water solubility, mg/L	0.056 (25 °C)	Recommended literature value	74,000 (20 °C)	Recommended literature value	0.5	EA (2009)
Soil-to-dust transport factor	0.5	EA 2009	0.5	EA 2009	1	EA (2009)

^a^ *NA* not applicable; the models usually do not require these values for organic/inorganic compounds

^b^ *NR* not relevant

^c^The experimentally determined value of *K*_AW_ for the Cl^−^ ionic concentration of 0.2 × 10^−3^ mol

^d^The value estimated from the soil/water partition coefficient for the inorganic components of mercury with the organic carbon content of 0.0348 (equivalent to 6 % of SOM)

**Table 6:** Models simulating mercury transport and transformation in aquatic system

Model	Properties	Survey site	Investigated media	Assessed forms of mercury
Coastal system				
ECoS (the Estuarine Contaminant Simulator model)	Estimation of contamination with Hg	Ria de Aveiro Coastal Lagoon (Portugal)	Suspended sediments, stagnant water	THg
2D STATRIM (the 2D STAtionary TRIeste gulf Mercur, a model with two submodels, 2D MIKE21MT, a model of transport in sediments, and PCFLOW2D-HD, a hydrodynamic model)	Simulations of Hg transport and transformation	The Gulf of Trieste	Suspended sediments, stagnant water, plankton	Hg^2+^, Hg^0^, MeHg, THg
Modified PCFLOW 3D (a hydrodynamic model containing a module for transport in sediments)	Simulations of Hg transport and transformation	The Gulf of Trieste	Suspended sediments, stagnant water	Hg^2+^, Hg^0^, MeHg, THg
Other				
EMMMA Environmental Mercury Mapping, Modeling, and Analysis	Determination of the characteristics of contamination with Hg, simulations of Hg transport and transformation, simulations of bioconcentration in fish	–	Suspended sediments, water, fish	Hg^2+^, Hg^0^, MeHg, THg

Source: Wang et al. (2004)

## Conclusion

The surveys on the mercury contents in oceanic and marine waters carried out in the second half of the twentieth century showed that its levels were comparable to those that now occur in the waters of the global ocean. This phenomenon relates to the fact that the mercury level in the global ocean has not yet reached the phase of a dynamic equilibrium with its level in the atmosphere. It follows from the following three major premises:

- The average residence time of mercury in oceanic waters is 20–30 years, whereas that in the atmosphere varies between 0.8 and 2 years; thus, the mercury discharged into the ocean is removed from there much more slowly than the mercury emitted into the atmosphere;
- Oceanic waters concentrate much larger masses of mercury of natural (geogenic) origin rather than those of anthropogenic origin, which are close to the masses originating from the preindustrial period; and
- A large part of the mercury which is vertically transported from the oceanic depths to the surface layers is transported back to the mixed layer, which is more active biologically.

The premises listed above indicate that an increase in the mercury concentration level in oceanic waters will be very slow and may take hundreds of years, even if an increase in the mercury concentration level in the atmospheric air is not observed (Selin et al. 2010; Mason et al. 2012). In 1990–1996, there was a significant reduction in the quantities of mercury and other dangerous substances discharged into oceanic waters. Since 1997, the quantities of mercury discharged into the oceans have remained at a relatively constant level.
